# Thirty Years of the Biology of Spermatozoa: The Rise and Future of an Evolutionary Paradigm

**DOI:** 10.1002/ece3.72985

**Published:** 2026-01-23

**Authors:** Leigh W. Simmons

**Affiliations:** ^1^ Centre for Evolutionary Biology, School of Biological Sciences (M092) The University of Western Australia Crawley Western Australia Australia

**Keywords:** cryptic female choice, gametic incompatibility, sperm competition, sperm form and function

## Abstract

In the early 1970s, Geoff Parker recognised that because females frequently mate with multiple males, competition for fertilizations will impose significant sexual selection on males and their ejaculates. Post‐mating sexual selection has since developed into a significant evolutionary paradigm, in no small part fostered by the biennial meeting of the Biology of Spermatozoa (BoS) community. BoS celebrated its thirtieth anniversary in 2025, prompting reflection on the development of the discipline and its future. The paradigm has shifted from one focused predominantly on competition among males for fertilizations to a holistic appreciation of the role of females and ova in determining fertilisation success, and the interacting effects of males and females on fertilisation outcomes. The field has transitioned from a phenotypic focus to one in which omics are yielding unprecedented insight into the biology of sperm, ova and male and female reproductive fluids that affect fertilisation dynamics. Understanding sperm biology has become a strongly interdisciplinary venture, fostered by the BoS meetings. Key discoveries over the last 30 years are highlighted and areas for future development identified. The history of this field highlights the critical role played by small, focused meetings and workshops.

## Graphical abstract

A sperm bundle taken from the seminal vescicles of the dung beetle, *Onthophagus taurus*. Image credit: Leigh W. Simmons.

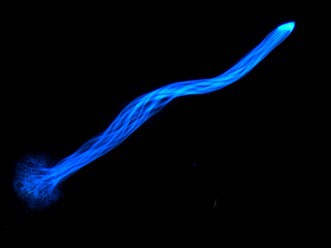

## 1 Introduction

Darwin (1871) defined sexual selection as ‘the advantage which certain individuals have over other individuals of the same sex and species, in exclusive relation to reproduction’, arguing that competition among males for access to females and female choice of mating partners could explain the evolution of exaggerated secondary sexual traits that otherwise seemed problematic for natural selection. While at the time contentious, sexual selection acting on traits that affect mating success is now widely recognised as a ubiquitous driving force of evolutionary change (Andersson 1994; Zuk and Simmons 2018). However, a significant advance in sexual selection theory came in the 1970s, when Parker (1970a) realised that, because females will often mate with more than one male, sexual selection would continue after mating.

Parker published two seminal papers in 1970. In the first, he demonstrated empirically how male yellow dungflies displace the sperm stored by females from their previous mates so that a male's fertilisation success rises rapidly during copula with diminishing returns (Parker 1970b). Parker developed the Marginal Value Theorem to model how males should invest in each mating to maximise their reproductive success, taking into account the costs of searching for additional females. The predicted optimal copula duration of 42 min was close to that observed under field conditions, leading Parker to conclude that the mating behaviour of male dungflies was shaped by sperm competition (Parker 1970b). He extended these observations in what became the foundation to the study of post‐mating sexual selection, arguing that much of insect behaviour and physiology could be explained in the light of sperm competition, from copula behaviour to the transfer of substances in the ejaculate that affect female receptivity to remating, and the passive phases that often occur before and after mating (Parker 1970a). Parker et al. (1972) made his second foundational contribution to post‐mating sexual selection when he showed theoretically how sperm competition could be responsible for the evolution of anisogamy with subsequent downstream effects on the evolution of the male–female phenomenon, what he later referred to as the sexual cascade (Parker 2014).

The evolutionary consequences of post‐mating sexual selection for reproductive biology of adult organisms and their gametes have developed into a mature and vibrant discipline within ecology and evolution, fostered in no small part by the biennial meetings of the Biology of Spermatozoa (BoS) community. The BoS celebrated its 30th anniversary in 2025, prompting reflection on key developments in the field and insights into its future.

## 2 A Brief Pre‐History

**FIGURE 1:**
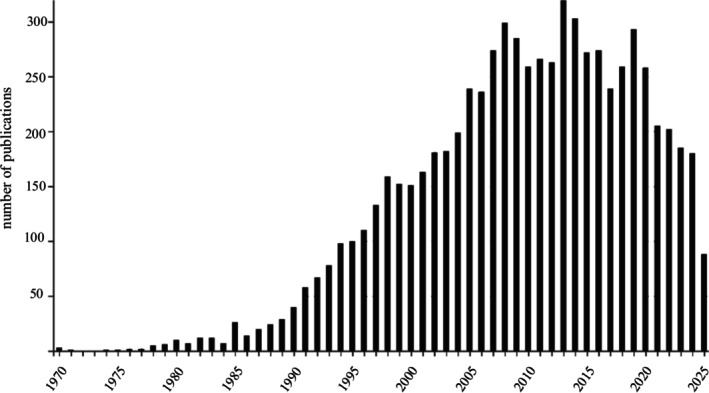
Number of publications by year derived from a Web of Knowledge search using the key word ‘sperm competition’ in August 2025.

The rise of the post‐mating sexual selection paradigm can be appreciated visually by plotting the numbers of publications with time (Figure 1). A collection of influential publications appeared in 1979. Waage (1979) reported the dual function of the damselfly penis in the removal of rival sperm prior to the delivery of his own sperm, Smith (1979) showed how repeated copulations by male water bugs ensured close to 100% paternity of the eggs a male incubates, Sivinski (1979) suggested that the astonishing morphological diversity in sperm form and function was likely due to gametic interactions, both through direct competition among sperm and via interactions with the female or her eggs, and Short (1979) suggested that testes size variation among the great apes could be explained by variation in mating systems and the extent of selection from sperm competition.

In 1983 Thornhill & Alcock published their influential synthesis of insect mating systems. In this book there were chapters reviewing the evolution via post‐mating sexual selection of mate guarding behaviour by males, the mechanisms of sperm competition and fertilisation in insects, and three chapters devoted to female agency, one on pre‐mating female choice, one on post‐mating cryptic female choice and one on the evolution of multiple mating by females (Thornhill and Alcock 1983). This volume consolidated the foundations on which the field of post‐mating sexual selection, and by extension sperm biology, would be built. Indeed, much of the pre‐history of sperm biology was dominated by those working with insects. This changed in 1987 when DNA fingerprinting technology revealed that high frequencies of extra‐pair paternity occurred in the nests of house sparrows (Burke and Bruford 1987; Wetton et al. 1987).

DNA fingerprinting technology opened the study of post‐mating sexual selection to vertebrates and generated a rapid growth in research during the coming decade. Parker published the first of his game theoretical models of sperm competition in 1990, predicting that sperm competition should favour the evolution of increased male expenditure on sperm production (Parker 1990a, 1990b), and Gage (1991) and Gage and Baker (1991) showed how male insects would increase the number of sperm in their ejaculate when copulating in the presence of a rival. These studies focused attention squarely on sperm and the evolutionary consequences of post‐mating sexual selection for sperm form and function. Geoff Parker had a large research group at the University of Liverpool, focused on the development of sperm competition theory and its empirical testing. Tim Birkhead also had a large research group at the University of Sheffield focused on sperm competition in birds, and he had the foresight to convene a small one‐day symposium for like‐minded people at the University of Sheffield in 1992 and again in 1993 for the exchange of ideas from researchers studying all aspects of sperm biology. The success of these symposia led to the first biennial Biology of Spermatozoa meeting in 1995, held at Losehill Hall in the Derbyshire Peak District. These small, regular and highly focused meetings have been instrumental in driving our increasing understanding of the evolutionary biology of spermatozoa.

## 3 Thirty Years of the Biology of Spermatozoa

The 1990s saw rapid developments in sperm biology research. In response to empirical findings that males would adjust the numbers of sperm in their ejaculate when mating in the presence of rivals (Gage 1991; Gage and Baker 1991), Parker extended his sperm competition games to predict how males should allocate sperm in response to the immediate risk and intensity of sperm competition. Parker's original models had predicted that as the risk of sperm competition (the probability of a female remating) increases, then the Evolutionary Stable Strategy ejaculate expenditure, measured as relative testis size, should increase, an effect expected across species and among populations within species (Parker 1990a, 1990b). His models of allocation per ejaculate predicted that males should initially increase the numbers of sperm ejaculated with increasing risk of sperm competition, but then decrease allocation with increasing sperm competition intensity (the number of males competing for a given clutch of eggs) because of diminishing returns on male allocation (Parker et al. 1996, 1997). Many contributions to the early BoS meetings sought to test and refine sperm competition game theory (Hosken and Stockley 1998; Pitnick and Karr 1996). The among species predictions have been largely borne out by comparative analyses of a range of taxonomic groups. Meta‐analysis of these comparative data, from insects and arachnids to mammals, yielded a general effect of risk on testes size in the region of 0.6, regardless of whether the analyses used behavioural or genetic estimates of the strength of sperm competition (Lüpold, de Boer, et al. 2020). Likewise, within species studies from a broad range of invertebrate and vertebrate species have shown that males will increase the numbers of sperm allocated to an ejaculate when they perceive an elevated risk of sperm competition in their environment (Kelly and Jennions 2011). However, the predictions for Parker et al. (1996) intensity models have received mixed support and the general effect size from meta‐analysis does not differ from zero (Kelly and Jennions 2011). An effort in understanding why this might be so seems justified. One reason might be that the intensity models were developed specifically for free spawning fishes, where males ejaculate simultaneously as a group, yet many tests of the predictions have come from studies of internally fertilising species that have a very different mating biology. We need more careful selection of appropriate study organisms when testing available theory, and we need new theory for strategic ejaculation in response to sperm competition intensity that seek predictions when fertilisation is internal and when matings occur sequentially rather than simultaneously as they do in free spawning external fertilisers.

In 1995 Eberhard provided an overview at BoS of his monograph on female agency in post‐mating sexual selection (Pitnick and Karr 1996), published 1 year later (Eberhard 1996). This volume had a significant impact on the study of sperm biology, focusing as it did on post‐mating sexual selection via cryptic female choice. It came as no surprise to those studying insects, as cryptic female choice had been well articulated by Thornhill and Alcock (1983) over a decade previously, and many entomologists had been studying female agency in post‐mating sexual selection for some time. But it was hugely influential in correcting a taxonomic bias, suggesting as it did any number of ways by which female vertebrates as well as invertebrates could, in theory, control which males fertilised their ova.

In his early work Parker (1970b) had assumed that female dungflies were passive, so that males simply ejaculated into a vessel displacing sperm from it at a constant rate. However, using males labelled with different radioactive isotopes, Simmons et al. (1999) showed how males in fact ejaculate into the bursa and that sperm are transferred to the spermatheca at a much slower rate, with the female's spermathecal ducts responsible for the uptake of sperm from where they are delivered by the male, a process later confirmed by histological observation (Hosken and Ward 2000). Parker and Simmons (2000) developed models of sperm displacement in which females were actively involved in the uptake of sperm, showing how a model with active females predicted the observed copula duration much better than one in which females were passive. Pizzari and Birkhead (2000) showed how female fowl eject ejaculate after mating, and are more likely to do so when mated to a subordinate male, and Snook and Hosken (2004) showed how *Drosophila* females adopted a similar post‐mating behaviour, dumping ejaculate from the reproductive tract shortly after the termination of copula. Female agency in sperm storage and use is now widely recognised as critical for our understanding of fertilisation biology (Firman et al. 2017), and studies of female effects in sperm biology, and of the biology of ova themselves, have come to share centre stage at contemporary BoS meetings (Rowe and Rosengrave 2020; Whittington and Ålund 2023). For example, in his plenary talk at BoS 2025, Luca Jovine from the Karolinska Institute reviewed our current understanding of egg‐sperm recognition proteins from molluscs to mammals, providing insight into the molecular mechanisms of sperm‐egg protein binding and fertilisation (Nishio et al. 2024).

An important development in the mid 1990s came in the form of two influential synthetic reviews by Zeh and Zeh (1996, 1997). They argued that polyandry was adaptive for females in that it provided them with post‐mating mechanisms for avoiding reproductive incompatibility. There are a variety of reasons why females might want to select among potential sires, from the avoidance of intragenomic conflict to the fertilisation of ova with compatible genotypes that enhance offspring performance. Zeh (1997) offered support for the latter with her work on pseudoscorpions, stimulating many studies that have since found strong effects of various forms of compatibility in selective fertilisation made possible by polyandry. For example, in *Drosophila melanogaster* the success of a particular male's sperm was found to depend on the genotype of the female with which he mates (Clark et al. 1999). In *D. pseudoobscura* , the selfish gene *Sex Ratio* kills Y‐bearing sperm biasing the offspring sex ratio toward females which favour its transmission. Sperm competition is expected to favour males without the selfish gene because *SR* carrying males have reduced sperm numbers and fertilisation success (Price, Bretman, et al. 2008). Polyandry should therefore be adaptive as it protects females from transmitting the fitness reducing *SR* gene to their offspring. As predicted, Price, Hodgson, et al. (2008) showed how populations of *D. pseudoobscura* with the SR gene evolved increased rates of polyandry. In their studies of guppies, Gasparini and Pilastro (2011) found that the sperm swimming velocity and subsequent fertilisation success of sperm from unrelated males was increased by the female's ovarian fluid to a greater extent than the sperm of brothers, facilitating the avoidance of incompatibility through inbreeding. Fitzpatrick et al. (2020) showed how, in vitro, male by female interaction effects contribute substantially to the attraction of human sperm to ova. As Craig Purchase discussed at BoS 2025, these types of gametic interactions can be important guards against hybridization between closely related species, such as salmonids that spawn in the same river systems (Yeates et al. 2013). Recent theoretical contributions have shown how such conspecific sperm precedence can be an important driver of speciation (Kustra et al. 2025).

An equally important advance in 1995 came from the finding that in *D. melanogaster* , seminal fluid can have negative impacts on female lifespan, contributing to the cost of mating for females (Chapman et al. 1995). This study highlighted how male adaptations to sperm competition may not be in the female's fitness interests. Indeed, Rice (1996) showed that when female evolution is arrested, males evolved to be more harmful to females while increasing their own fitness. These two studies influenced the study of sperm biology in 3 significant ways. First, they drew attention to the importance of sexual conflict over male and female reproductive interests, the theoretical foundations of which had been laid down by Parker (1979) nearly two decades previously. Second, they generated an increased focus on non‐sperm components of the ejaculate, and third, Rice's (1996) paper stimulated a flurry of studies using experimental evolution in the study of sperm biology.

Experimental evolution as a technique has generated considerable insight into sperm biology. Using experimentally evolved populations of *D. melanogaster* derived from Rice's original lines, Pitnick et al. (2001) showed how after 61 and 81 generations of experimental evolution respectively, testis size and the number of sperm were both greater in populations evolving with post‐mating sexual selection compared with those evolving under enforced monogamy. Similar findings came from Hosken and Ward's (2001) study of yellow dungflies, whereby males from polyandrous lines had larger testes and greater competitive fertilisation success than males from monogamous lines after just 10 generations of experimental evolution. These findings have been extended to mammals, with male house mice evolving under polyandry having increased sperm numbers and motility (Firman and Simmons 2010b), and increased competitive fertilisation success (Firman and Simmons 2010a) compared with males from populations evolving under enforced monogamy. Experimental evolution has thus confirmed the cause‐and‐effect relationship between sperm competition and sperm production that correlational comparative analyses had suggested. Moreover, in their study of house mice, Firman et al. (2014) showed correlated evolutionary responses in females to post‐mating sexual selection, specifically in the defensiveness of ova to fertilisation potentially mediated by changes in the extracellular matrix of cumulus cells surrounding the ova (Keeble et al. 2021).

The importance of females in mediating sperm evolution was exemplified by Miller and Pitnick's (2002) work on the coevolution of sperm and female reproductive tract morphology. They artificially selected lines of *D. melanogaster* for increasing and decreasing seminal receptacle length or increasing and decreasing sperm length, affecting rapid changes in phenotype. They then competed males with long or short sperm within females with long or short seminal receptacles, finding that males with longer sperm had a fertilisation advantage in competition, particularly when competing within long seminal receptacles. Importantly, they found that the brothers of females selected for long or short seminal receptacles had long or short sperm respectively, demonstrating the correlated evolution of sperm and female reproductive tract morphology (Miller and Pitnick 2002). To understand why sperm length and seminal receptacle length should exhibit patterns of coevolution, Manier et al. (2010) created transgenic flies that have either green or red fluorescing sperm heads to reveal the mechanism of sperm displacement in real time. As in yellow dungflies, the male was shown to ejaculate into the bursa copulatrix. Sperm from the first and second male then swim into and out of the seminal receptacle, with an increasing proportion of sperm from the second male becoming resident in the seminal receptacle and an increasing proportion of sperm from the first male present in the bursa copulatrix. The female puts an end to this displacement by dumping sperm from the bursa, containing predominantly first male sperm (Manier et al. 2010). Longer sperm have an advantage in entering and remaining within the seminal receptacle (Lüpold et al. 2012), but why should this be so? We need mechanistic studies of sperm traits to fully appreciate the selection pressures acting on spermatozoa form and function. Two such studies were presented at BoS 2025. In her plenary, Lisa Fauci explained how the fluid dynamics of flexible filaments affect the movements of flagellate sperm within the confines of a narrow tube, and so how flagella length determines a sperm's ability to swim effectively within the narrow confines of the female reproductive tract (Morshed et al. 2024). Jasmin Alsous showed how *D. melanogaster* sperm, at ~1.8 mm in length, manage to swim effectively without becoming entangled. These giant sperm exhibit extraordinary collective behaviour, leveraging on each other to gain collective progressive motility, within both male and female reproductive tracts, and so avoid entanglement (Alsous et al. 2025). These mechanistic findings help explain the male × male, male × female and male × male × female interaction effects on sperm storage and subsequent fertilisation outcomes (Lüpold, Reil, et al. 2020). Strides are now being made in uncovering the macroevolutionary patterns of sperm‐seminal receptacle length coevolution among species of *Drosophila* and their genomic underpinnings, shedding light on the mechanisms of sexual selection responsible for one of nature's most exaggerated sexual traits (Syed et al. 2025).

During the mid‐2000s there was a distinct change in the focus of research on post‐mating sexual selection. There was an increased emphasis on the role of seminal fluids in sperm biology and a marked shift in the level of analysis from phenotypic to genomic and proteomic. These developments no doubt arose in no small part to work on the discovery and function of seminal fluid proteins in *Drosophila* (Wolfner 1997), from genomic work on the evolutionary divergence of gamete recognition proteins in broadcast spawning invertebrates (Vacquier 1998), and the discovery that male reproductive genes are evolving rapidly among the great apes in close association with the strength of selection from sperm competition (Wyckoff et al. 2000).

We have learnt much about the function and evolution of seminal fluid proteins from studies of *Drosophila* (Wigby et al. 2020). *Drosophila* are excellent models because of the extraordinary genetic resources that are available with which to tease apart the function of novel seminal fluid proteins (sfps). But as powerful as *Drosophila* may be as a study system, we need to examine these phenomena in a broader array of organisms in order to appreciate their general role in sperm biology. Given the lack of genomic resources, the study of seminal fluid effects on sperm biology in non‐model organisms was made first at the phenotypic level. For example, Cornwallis and O'Conner (2009) showed how in jungle fowl, seminal fluid affects the swimming velocity of sperm and that males adjust sperm swimming speed in response to female quality, presumably via their seminal fluids because sperm placed in seminal fluid produced by males mating to small‐combed females swam slower than did sperm placed into seminal fluid produced by males mating to large‐combed females. The discovery of novel sfps in non‐model species requires considerable efforts using proteomic, transcriptomic and genomic approaches. In their study of macrostomum flatworms, Patlar et al. (2020) identified a suit of putative sfp gene sequences and tested their functional significance using RNA interference. They identified at least two sfps, suckless 1 and suckless 2, that when knocked down in donor worms result in recipient worms being more likely to suck out transferred ejaculate. Similarly, Simmons and Lovegrove (2023) identified a group of eight sfps in the cricket *Teleogryllus oceanicus* , four of which when knocked down reduced the competitive fertilisation success of males, and one protein, gagein, was found to be a strong predictor of competitive fertilisation success in unmanipulated competing males. We need many more studies such as these, across a broad array of taxa to fully appreciate the complexity of sperm‐seminal fluid dynamics, the roles of sfps in male fertility, and their positive and negative effects on females and resulting offspring.

Given the success of sperm competition game theory in predicting male expenditure on sperm, it is perhaps surprising that less theory has been developed around the problem of sfp expenditure. Perhaps this is because sfps can have many different functions, making general prediction of male allocation difficult to formulate. Nevertheless, some effort has been made in this area (Cameron et al. 2007; Dhole and Servedio 2014; Alonzo and Pizzari 2010). Empirical studies that have looked for strategic allocation of sfps are similarly rare. Leading the way as always, work with *D. melanogaster* has shown how males will increase their allocation of both ovulin (a sfp that stimulates egg production) and sex peptide (a sfp that reduces a female's propensity to remate) when mating in the presence of rival males (Wigby et al. 2009). They will also adjust their allocation of sfps in response to a female's mating status, reducing their expenditure on ovulin when mating with non‐virgin females, a response predicted because previous males will have already stimulated a female to lay eggs making the donation of yet more ovulin redundant (Sirot et al. 2011). Work on *T. oceanicus* has shown how males exposed to potential rivals will increase expenditure on a number of sfps that affect sperm viability, competitive fertilisation success and female receptivity (Simmons and Lovegrove 2017, 2023; Moschilla et al. 2020). While in house mice, males exposed to rival males increase the abundance of three seminal vesicle secreted proteins in their ejaculate (Ramm et al. 2015). Much work is needed in this area, particularly since seminal fluid can contain hundreds of different proteins, and different proteins can have different functions making general predictions about male responses to sperm competition difficult.

## 4 Future Directions

Looking at the distribution of published papers in Figure 1 might suggest that sperm biology has seen its day and is in decline. However, it was clear from the contributions at BoS 2025 that the discipline remains vibrant. The dip in publication output likely reflects more the trials and tribulations of COVID as there is still much we don't understand about sperm biology. Strides are being made in uncovering the genetic basis underlying gametic incompatibility (Yasir Ahmed‐Braimah, BoS 2025 communication). New areas are developing around the role of the microbiome in male fertility and competitive fertilization success (Stephan Lüpold, BoS 2025 communication, Maggu et al. 2025, Rowe et al. 2020, McNamara et al. 2024), and the impacts of heat stress and other abiotic impacts of climate change on male fertility (Steve Ramm, BoS 2025 communication, Walsh et al. 2019). Here I highlight four areas that are set to offer particularly unique insights into our understanding of the evolutionary biology of spermatozoa.

We know that female reproductive fluids are important in affecting sperm function. Work on mussels, *Mytilus galloprovincialis* , for example has shown how egg chemoattractants affect the swimming performance and attraction of compatible sperm for fertilisation (Lymbery et al. 2017). In fishes, ovarian fluid has been shown to enhance the paternity success of territorial or parental males over those adopting alternative ‘sneaker’ mating tactics (Alonzo et al. 2016; Pinzoni et al. 2023). But how? We have developed considerable knowledge about the proteins in the seminal fluids of males and their function, and we need similar approaches to discover the proteins in female reproductive fluids that affect sperm function. Work on *D. melanogaster* suggests this would be a fruitful endeavour; the female reproductive tract proteome shows profound changes in protein composition following mating, with these changes potentially affecting sperm function (McDonough‐Goldstein et al. 2021).

Another area ripe for development revolves around non‐genetic components of sperm and their function. We now know that non‐genetic paternal effects can be transmitted to offspring, influencing phenotypes from behaviour to physiology, via small RNAs packaged in sperm (Immler 2018; Evans et al. 2019; Yin et al. 2025). Exosomes in the *Drosophila* seminal fluid also carry small RNAs that are translated by females in their reproductive tract (Matzkin et al. 2024, Matzkin BoS 2025 communication). There is evidence from *Drosophila* that the abundance of some microRNAs in sperm can be increased by an evolutionary history of sperm competition, begging the question as to their role in sperm function (Hotzy et al. 2022). Do RNAs in sperm affect their motility and fertilisation capacity? Can males upregulate sperm RNA function in response to the risk of sperm competition, and what are the effects of these RNAs on the performance of zygotes and offspring? And might sperm RNAs be involved in the phenotypic plasticity of sperm function in response to female reproductive fluids?

The established dogma is that mature ejaculated sperm are transcriptionally silent. This is because, in mammals, histones are replaced by protamines and DNA is both folded and tightly packed. Further, during maturation, sperm cytoplasm is greatly reduced providing little space for transcription (reviewed in Lymbery et al. 2025). However, there is growing, albeit circumstantial, evidence to suggest that DNA transcription might occur in mature ejaculated sperm (Lymbery et al. 2025). Gene expression in mature sperm could provide mechanistic answers to the phenotypic responses of sperm to female reproductive fluids, for interactions between sperm and ova and for variation in the subsequent performance of offspring. A concerted effort is required to discover whether sperm are, or are not, transcriptionally silent.

Finally, we have much to learn about the extraordinary divergence in sperm morphology across the animal tree of life. We know from comparative analyses that sperm length generally increases in response to sperm competition (Lüpold, de Boer, et al. 2020) and that evolutionary transitions to internal fertilisation are associated with increased sperm length (Kahrl et al. 2021). Interactions with the female reproductive tract are clearly involved in sperm evolution, as demonstrated by the evolution of sperm length in *Drosophila* (Miller and Pitnick 2002) and other taxa (Pitnick et al. 2009). But variation in length trivialises the true complexity of sperm morphology.

How exactly does post‐mating sexual selection act on aspects of sperm morphology other than length? Marie‐Orleach et al. (2024) recently quantify episodes of both pre‐ and post‐mating sexual selection acting on adult macrostomum flatworms, their genitalia and their gametes. They used a transgenic line of the transparent flatworm expressing green fluorescent protein in all cell types, including sperm cells, enabling in vivo sperm tracking. For sperm traits, they found that individuals with sperm having a longer terminal brush had a greater reproductive success. Moreover, a composite trait that contrasts the length of the sperm body with the length of the stiff lateral bristles was associated with the greatest reproductive success. In this species we know that the lateral bristles aid donor sperm in anchorage in the reproductive tract when a recipient attempts to suck ejaculate out after copulation (Schärer et al. 2011). We need many more studies like this, of selection acting on the morphological components of sperm for different species, because, unlike sperm length, the extraordinary variation in sperm morphology across the animal tree of life is unlikely to be explained by a single, general phenomenon.

The biennial BoS meetings are firmly grounded in evolutionary biology. They focus on the evolutionary biology of reproduction generally, from the behaviour and morphology of the whole organism to its gametes. As such, research on the evolution of genital morphology, for example, has been a regular feature of BoS meetings (e.g., Brennan et al. 2010; Schultz et al. 2016). By taking an evolutionary approach we have discovered much about the biology of spermatozoa themselves, and the BoS meetings have proved a significant driver of those discoveries (Birkhead et al. 2009). Understanding how post‐mating sexual selection drives the evolution of sperm form and function requires insight into mechanisms (Simmons and Siva‐Jothy 1998), Tinbergen's (1963) how and why need to be addressed together. By engaging with researchers in mechanistic disciplines such as cell biology, genomics and proteomics, the BoS meetings have fostered interdisciplinary collaborations that have fast‐tracked our understanding of the evolution of nature's most diverse cell type. Parker (2007) famously said that the BoS was by far his most favourite meeting in the academic calendar, a sentiment shared by many of us who have contributed to the Biology of Spermatozoa over its 30 year history.

## Author Contributions

**Leigh W. Simmons:** conceptualization (lead), writing – original draft (lead).

## Conflicts of Interest

The author declares no conflicts of interest.

## Data Availability

The author has nothing to report.
